# Influence of Baseline HbA1c and Antiplatelet Therapy on 1-Year Vein Graft Outcome

**DOI:** 10.1016/j.jacasi.2021.11.009

**Published:** 2022-03-15

**Authors:** Yunpeng Zhu, Junlong Hu, Minlu Zhang, Qing Xue, Hao Liu, Rui Wang, Xiaowei Wang, Zhaoyun Cheng, Qiang Zhao

**Affiliations:** a Department of Cardiovascular Surgery, Ruijin Hospital Shanghai Jiao Tong University School of Medicine, Shanghai, China; b Department of Cardiovascular Surgery, Henan Provincial People's Hospital Heart Center, Central China Fuwai Hospital of Zhengzhou University, Zhengzhou, China; c Department of Cancer Control and Prevention, Shanghai Municipal Center for Disease Control and Prevention, Shanghai, China; d Department of Cardiovascular Surgery, Changhai Hospital of Shanghai, Shanghai, China; e Department of Cardiothoracic Surgery, Xinhua Hospital Shanghai Jiao Tong University School of Medicine, Shanghai, China; f Department of Cardiovascular Surgery, Nanjing First Hospital, Nanjing Medical University, Nanjing, China; g Department of Cardiovascular Surgery, Jiangsu Province Hospital, Nanjing, China

**Keywords:** aspirin, coronary artery bypass grafting, glycated hemoglobin A, patency, ticagrelor, vein graft, CABG, coronary artery bypass graft, CAD, coronary artery disease, CI, confidence interval, DM, diabetes mellitus, HbA1c, glycated hemoglobin, ITT, intention to treat, MACE, major adverse cardiovascular event, OR, odds ratio, T+A, ticagrelor plus aspirin

## Abstract

**Background:**

The influence of baseline HbA1c levels on vein graft outcomes post coronary artery bypass grafting (CABG) remains unclear.

**Objective:**

The purpose of this study was to assess the association between baseline HbA1c and 1-year vein graft patency, and the effects of antiplatelet therapy on the 1-year vein graft patency after CABG in patients with baseline HbA1c <6.5% vs ≥6.5%.

**Methods:**

We examined the subgroups with baseline HbA1c <6.5% vs ≥6.5% from the DACAB trial (NCT02201771), in which 500 patients were randomly allocated to receive ticagrelor plus aspirin (T+A), ticagrelor alone (T), or aspirin alone (A) for 1 year after CABG. The primary outcome was the vein graft patency (FitzGibbon grade A) at 1 year.

**Results:**

A total of 405 patients with available baseline HbA1c data were included in this subgroup analysis. Of them, there were 233 patients (678 vein grafts) with baseline HbA1c <6.5% and 172 patients (512 vein grafts) with baseline HbA1c ≥6.5%. Compared with the HbA1c <6.5% subgroup, the HbA1c ≥6.5% subgroup showed worse 1-year vein graft patency (adjusted odds ratio [OR] for nonpatency: 1.69, 95% confidence interval [CI]: 1.08-2.64). T+A showed higher vein graft patency than A in both HbA1c <6.5% (adjusted OR for nonpatency: 0.34, 95% CI: 0.15-0.75) and HbA1c ≥6.5% subgroups (adjusted OR for nonpatency: 0.45, 95% CI: 0.19-1.09), without an interaction effect (*P* for interaction = 0.335), whereas T did not show more significant improvement than A in both subgroups.

**Conclusions:**

In the DACAB trial, lower baseline HbA1c was associated with higher vein graft patency 1 year after CABG. T+A improved 1-year vein graft patency vs A, irrespective of baseline HbA1c.

Diabetes mellitus (DM) is associated with a worse prognosis of coronary artery diseases (CAD).^1^ A large study of 1.9 million people showed that type 2 diabetes was positively associated with the incidence of stable angina and myocardial infarction.^2^ Approximately two-thirds of the deaths in patients with DM are attributable to CAD,^1^ and CAD occurs approximately 15 years earlier in patients with DM than those without.^3^ A systematic review indicated that 32.2% of patients with DM have CAD.^4^ Approximately 70% of the patients 65 years or older and with DM will die of CAD or stroke.^1^

For diabetic patients with 1- or 2-vessel disease, including the proximal left anterior descending artery, 3-vessel disease, or left main coronary artery disease, the current European guidelines made a Class I recommendation on coronary artery bypass grafting (CABG) as the gold standard for myocardial revascularization.^5^ The internal mammary artery graft is often considered the first choice because of its higher 10-year patency rate (>90%) and improved survival compared with any other grafts.^6^ Other arterial grafts (especially the radial artery) should be preferred as the second choice.^7^ Nevertheless, the saphenous vein grafts remain the most commonly used graft worldwide, with occlusion rates of 11% within 1 year after surgery and 40% to 50% at 10 years.^8^

Although blood glucose levels are punctual and vary greatly within a given day, glycated hemoglobin (HbA1c) levels represent the glucose levels over the past 90 days and can be used to evaluate glycemic control.^9^ The DCCT (Diabetes Control and Complications Trial) showed that high HbA1c levels were a predictor of microvascular complications of DM and that maintaining HbA1c at <7% decreased the occurrence of diabetic retinopathy, nephropathy, and neuropathy, as well as cardiovascular risk and mortality.^10^, ^11^, ^12^ Recently, several studies revealed that higher preoperative HbA1c levels were associated with a higher cardiovascular risk and long-term mortality after CABG, irrespective of DM.^13^, ^14^, ^15^ However, the effect of HbA1c control on vein graft outcome is still unclear, especially in the early term.

Therefore, this post hoc subgroup analysis of the DACAB (Different Antiplatelet Therapy Strategy After Coronary Artery Bypass Graft Surgery) trial aimed to assess the effects of ticagrelor with or without aspirin vs aspirin alone on the 1-year vein graft patency after CABG according to baseline HbA1c levels. The results could provide additional data for the personalization of antiplatelet therapy after CABG.

## Methods

### Data source

The DACAB trial (NCT02201771) was a multicenter, open-label, randomized trial of ticagrelor with or without aspirin vs aspirin alone that enrolled patients undergoing elective CABG (75% off-pump) in China.^16^ Briefly, it was a randomized, multicenter, open-label trial that enrolled 500 patients scheduled to undergo elective CABG surgery at 1 of 6 hospitals in China. The eligible patients were randomized 1:1:1 to receive antiplatelet therapy within 24 hours after CABG and for 1 year: 1) ticagrelor (90 mg twice daily) plus aspirin (100 mg once daily); 2) ticagrelor alone (90 mg twice daily); or 3) aspirin alone (100 mg once daily). Lipid-lowering and antidiabetic drugs were at the discretion of their treating physicians. For the present post hoc analysis, baseline HbA1c levels were available for only 405 participants; hence 95 participants were excluded. The patients were grouped according to baseline HbA1c (<6.5% and ≥6.5%).^17^

The present analysis was approved by the ethics committee of the Ruijin Hospital Shanghai Jiao Tong University School of Medicine. All participants or their legal representatives provided written informed consent before study enrollment. This consent included the post hoc analyses of the trial data.

### Primary outcome/assessment

The outcome of the grafts was assessed by computed tomography angiography or coronary angiography and graded as described by FitzGibbon et al.^18^ The primary outcome was defined as patency (FitzGibbon grade A) vs nonpatency (FitzGibbon grade B+O).

### Statistical analysis

All 405 patients (intent-to-treat [ITT] population) with 1,190 vein grafts from the original DACAB trial and available baseline HbA1c data were included in the present analysis. In the ITT analysis, all grafts with missing outcome assessments were considered FitzGibbon grade O.

Continuous variables were presented as means ± standard deviations or medians with interquartile ranges. Differences in baseline characteristics between treatment groups within baseline HbA1c <6.5% vs ≥6.5% and among subgroups were compared using the analysis of variance test. Categorical variables were presented as numbers and percentages and were analyzed using the chi-square test or Fisher’s exact test. The analyses were performed on a per-graft and per-patient basis. In the per-graft analysis, as each patient received multiple vein grafts, a generalized estimating equation model was used to analyze the repeated measures of outcomes. A logit link function was applied, and an independent covariance structure was used to model the correlation of the responses from the same patients. The stability of the model was verified using unstructured and exchangeable covariance structures. The estimation of the between-subgroup differences in vein graft patency was presented as odds ratios (ORs) and 95% confidence intervals (CIs). In the per-patient analysis, the patient outcome was classified according to the worst graft outcome in the patient. Logistic regression was used to obtain ORs and corresponding 95% CIs. Confounders were selected based on data availability and the literature, including age, sex, medical history of hypertension and hyperlipidemia, SYNTAX score, target vessel distribution, antiplatelet therapy, and statin use at 1-year after CABG. The consistency of the treatment effect among the subgroups was explored using a treatment × subgroup interaction. An HbA1c cutoff point of 6.5% was used in the analyses. Besides, HbA1c cutoff point of 7%, 7.5%, and 8% was used as sensitive analysis.

A 2-sided level of significance of 0.05 was applied. All analyses were performed using SAS 9.4 (SAS Institute).

## Results

### Characteristics of the patients

According to available baseline HbA1c data, there are 233 patients (678 vein grafts) in the <6.5% subgroup and 172 patients (512 vein grafts) in the ≥6.5% subgroup (Table 1). The baseline characteristics were generally comparable between the baseline HbA1c <6.5% vs ≥6.5% subgroups, except for sex (85.0% male in HbA1c <6.5% subgroup vs 76.7% male in ≥6.5% subgroup, *P* = 0.035) and statin use at 1 year post CABG (98.3% in HbA1c <6.5% subgroup vs 93.0% in ≥6.5% subgroup, *P* = 0.007). Supplemental Table 1 presents the characteristics of the patients according to baseline HbA1c <6.5% vs ≥6.5% and antiplatelet treatment.

**Table 1:** Characteristics of the Patients Between Baseline HbA1c ≥6.5% and <6.5%

	HbA1c <6.5% (n = 233)	HbA1c ≥6.5% (n = 172)	*P* Value
Age, y	63.8 ± 8.1	64.2 ± 8.0	0.622
Male	198 (85.0)	132 (76.7)	0.035
Clinical status			
Stable angina	80 (34.3)	71 (41.3)	0.321
Unstable angina	146 (62.7)	95 (55.2)	
NSTEMI	7 (3.0)	6 (3.5)	
NYHA functional class III-IV	89 (38.2)	63 (36.6)	0.671
Medical history			
Myocardial infarction	77 (33.1)	54 (31.4)	0.725
Stroke	32 (13.7)	20 (11.6)	0.531
Hypertension	173 (74.3)	135 (78.5)	0.323
Hyperlipidemia^a^	166 (71.2)	123 (71.5)	0.953
Peripheral vascular disease	43 (18.5)	34 (19.8)	0.739
Smoking	127 (54.5)	77 (44.8)	0.053
COPD	22 (9.4)	12 (7.0)	0.377
LVEDD, mm	49 (46, 53)	49 (46, 52)	0.206
LVEF, %	63 (57, 68)	62 (55, 68)	0.649
SYNTAX score			
Low (0-22)	33 (14.2)	21 (12.2)	0.767
Intermediate (23-32)	137 (58.8)	100 (58.1)	
High (≥33)	63 (27.0)	51 (29.6)	
EuroSCORE			
Low (0-2)	99 (42.5)	74 (43.0)	0.559
Medium (3-5)	104 (44.6)	70 (40.7)	
High (≥6)	30 (12.9)	28 (16.3)	
Medication use, baseline			
Beta blocker	213 (91.4)	160 (93.0)	0.553
ACEI/ARB	150 (64.4)	110 (64.0)	0.930
Statins	226 (97.0)	163 (94.8)	0.255
Medication use, 1 y			
Beta blocker	220 (94.4)	162 (94.2)	0.920
ACEI/ARB	121 (51.9)	100 (58.1)	0.215
Statins	229 (98.3)	160 (93.0)	0.007
Surgical procedure			
Pump use	35 (15.0)	32 (18.6)	0.337
Total grafts, n	877	657	-
Mean grafts/patient, n	3.8	3.8	-
IMA use	192 (82.4)	144 (83.7)	0.727

Values are mean ± SD, n (%), median (Q1, Q3), or n.

ACEI = angiotensin-converting enzyme inhibitor; ARB = angiotensin receptor blocker; COPD = chronic obstructive pulmonary disease; EuroSCORE = logistic European System for Cardiac Operative Risk Evaluation; HbA1c = glycated hemoglobin; IMA = Internal mammary artery; LVEDD = left ventricular end-diastolic diameter; LVEF = left ventricular ejection fraction; NSTEMI = non-ST-segment elevation myocardial infarction; NYHA = New York Heart Association; SYNTAX = Synergy Between Percutaneous Coronary Intervention With Taxus and Cardiac Surgery.

a Defined as baseline low-density lipoprotein cholesterol ≥1.8 mmol/L.

### Graft outcome between the baseline HbA1c subgroups

In the per-graft analysis, the 1-year vein graft patency (FitzGibbon grade A) and nonocclusion (FitzGibbon grade A+B) rates were 86.1% and 88.9% in the HbA1c <6.5% subgroup and 77.9% and 81.6% in the HbA1c ≥6.5% subgroup, respectively. Compared with the HbA1c <6.5% subgroup, the HbA1c ≥6.5% subgroup showed worse patency (adjusted OR for nonpatency: 1.69; 95% CI: 1.08-2.64; *P* = 0.021) and occlusion (adjusted OR for occlusion: 1.70; 95% CI: 1.04-2.79; *P* = 0.034). Similar results were observed for vein graft patency in the per-patient analysis (Table 2, Central Illustration). The results remained consistent in the sensitivity analyses using HbA1c <7.0% vs ≥7.0% (Supplemental Table 2), HbA1c <7.5% vs ≥7.5% (Supplemental Table 3), and HbA1c <8.0% vs ≥8.0% (Supplemental Table 4). When analyzed as a continuous variable, baseline HbA1c was associated with vein graft failure (for nonpatency per graft: adjusted OR: 1.25; 95% CI: 1.08-1.45; *P* = 0.003; for occlusion per graft: adjusted OR: 1.25; 95% CI: 1.07-1.46; *P* = 0.004) (Table 3).

**Table 2:** One-Year Vein Graft Outcome Between Baseline HbA1c ≥6.5% and <6.5%

1-Year Outcome	HbA1c <6.5%	HbA1c ≥ 6.5%	HbA1c ≥6.5% vs. <6.5% Adjusted OR (95% CI)	*P* Value
Per graft	n = 678	n = 512		
FitzGibbon grade A	584 (86.1)	399 (77.9)		
FitzGibbon grade B	19 (2.8)	19 (3.7)		
FitzGibbon grade O	75 (11.1)	94 (18.4)		
Patency (A)	584 (86.1)	399 (77.9)	1.69 (1.08–2.64)^a^	0.021
Nonocclusion (A+B)	603 (88.9)	418 (81.6)	1.70 (1.04–2.79)^b^	0.034
Per patient	n = 233	n = 172		
FitzGibbon grade A	177 (76.0)	113 (65.7)		
FitzGibbon grade B	10 (4.3)	13 (7.6)		
FitzGibbon grade O	46 (19.7)	46 (26.7)		
Patency (A)	177 (76.0)	113 (65.7)	1.62 (1.01–2.60)^a^	0.048
Nonocclusion (A+B)	187 (80.3)	126 (73.3)	1.41 (0.86–2.32)^b^	0.174

Values are n (%) unless otherwise noted.

CI = confidence interval; HbA1c = glycated hemoglobin; OR = odds ratio, OR was adjusted for age, sex, medical history of hypertension and hyperlipidemia, SYNTAX score, target vessel distribution, antiplatelet therapy, and statin use at 1 year after coronary artery bypass graft; SYNTAX = Synergy Between Percutaneous Coronary Intervention With Taxus and Cardiac Surgery.

a With nonpatency (B+O) as outcome.

b With occlusion (O) as outcome.

**Central Illustration:**
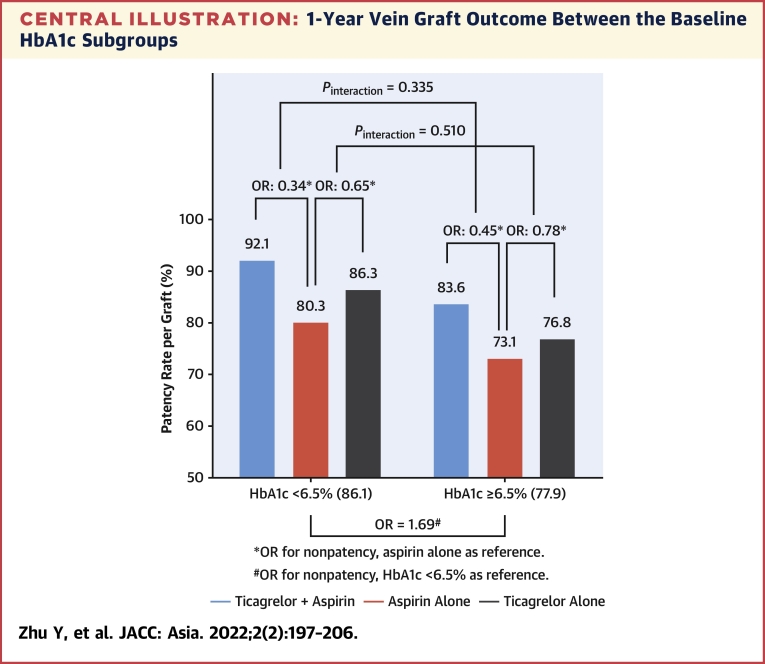
1-Year Vein Graft Outcome Between the Baseline HbA1c Subgroups Odds ratio was adjusted for age, sex, medical history of hypertension and hyperlipidemia, SYNTAX score, target vessel distribution, antiplatelet therapy and statin use at 1-year after coronary artery bypass graft. HbA1c = glycated hemoglobin.

**Table 3:** Baseline HbA1c Treated as Continuous Variable and Vein Graft Outcome 1 Year After CABG

1-Year Outcome	Patency (FitzGibbon Grade A)		Nonocclusion (FitzGibbon Grade A+B)	
	OR Adjusted for Nonpatency (95% CI)	*P* Value	OR Adjusted for Occlusion (95% CI)	*P* Value
Per graft	1.25 (1.08–1.45)	0.003	1.25 (1.07–1.46)	0.004
Per patient	1.29 (1.10–1.52)	0.002	1.26 (1.07–1.48)	0.007

CI = confidence interval; HbA1c = glycated hemoglobin; OR = odds ratio, OR was adjusted for age, sex, medical history of hypertension and hyperlipidemia, SYNTAX score, target vessel distribution, antiplatelet therapy, and statin use at 1 year after coronary artery bypass graft; SYNTAX = Synergy Between Percutaneous Coronary Intervention With Taxus and Cardiac Surgery.

We also analyzed artery graft outcome between the baseline HbA1c <6.5% vs ≥6.5% subgroups. In the per-graft analysis, the 1-year artery graft patency (FitzGibbon grade A) and nonocclusion (FitzGibbon grade A+B) rates were 96.9% and 97.4% in the HbA1c <6.5% subgroup and 97.7% and 98.5% in the HbA1c ≥6.5% subgroup, respectively. There was no difference in artery graft outcomes between the baseline HbA1c <6.5% vs ≥6.5% subgroups (all *P* > 0.05) (Supplemental Table 5).

### Vein graft outcome among randomized antiplatelet treatments in the HbA1c <6.5% and ≥6.5% subgroups

In the per-graft analysis, the 1-year vein graft patency rates were 88.5% with ticagrelor plus aspirin (T+A), 81.8% with ticagrelor alone, and 77.4% with aspirin alone. T+A had lower odds of nonpatency compared with aspirin alone in the HbA1c <6.5% subgroup (adjusted OR for nonpatency: 0.34; 95% CI: 0.15-0.75) and in the HbA1c ≥6.5% subgroup (adjusted OR for nonpatency: 0.45; 95% CI: 0.19-1.09). There was no interaction effect between the baseline HbA1c status and antiplatelet treatment (interaction *P* = 0.335). There were no significant differences in patency for ticagrelor alone vs aspirin alone in both HbA1c subgroups (all *P* > 0.05) and no interaction effect (interaction *P* = 0.510). Similar results were observed in the per-patient analysis (Table 4).

**Table 4:** 1-Year Vein Graft Outcome Among Randomized Antiplatelet Treatments in Baseline HbA1c Subgroups

1-Year Patency	T+A		T		A		T+A vs A		T vs A	
	n	n (%)	n	n (%)	n	n (%)	Adjusted OR^a^ (95% CI)	Interaction *P* Value	Adjusted OR^a^ (95% CI)	Interaction *P* Value
Per graft										
HbA1c<6.5%	229	211 (92.1)	211	182 (86.3)	238	191 (80.3)	0.34 (0.15-0.75)	0.335	0.65 (0.30-1.40)	0.510
HbA1c≥6.5%	171	143 (83.6)	185	142 (76.8)	156	114 (73.1)	0.45 (0.19-1.09)		0.78 (0.36-1.70)	
Per patient										
HbA1c<6.5%	79	67 (84.8)	73	55 (75.3)	81	55 (67.9)	0.43 (0.19-0.99)	0.973	0.77 (0.35-1.69)	0.269
HbA1c≥6.5%	59	46 (78.0)	60	35 (58.3)	53	32 (60.4)	0.42 (0.16-1.10)		1.03 (0.44-2.42)	

1-Year Nonocclusion	T+A		T		A		T+A vs A		T vs A	
	n	n (%)	n	n (%)	n	n (%)	Adjusted OR^b^ (95% CI)	Interaction *P* Value	Adjusted OR^b^ (95% CI)	Interaction *P* Value
Per graft										
HbA1c<6.5%	229	213 (93.0)	211	188 (89.1)	238	202 (84.9)	0.40 (0.17-0.94)	0.664	0.69 (0.29-1.66)	0.154
HbA1c≥6.5%	171	147 (86.0)	185	150 (81.1)	156	121 (77.6)	0.49 (0.18-1.31)		0.79 (0.34-1.84)	
Per patient										
HbA1c<6.5%	79	68 (86.1)	73	60 (82.2)	81	59 (72.8)	0.52 (0.22-1.21)	0.818	0.67 (0.29-1.57)	0.620
HbA1c≥6.5%	59	49 (83.1)	60	40 (66.7)	53	37 (69.8)	0.51 (0.18-1.45)		1.19 (0.49-2.92)	

A = aspirin; CI = confidence interval; HbA1c = glycated hemoglobin; OR = odds ratio, OR was adjusted for age, sex, medical history of hypertension and hyperlipidemia, SYNTAX score, target vessel distribution, antiplatelet therapy and statin use at 1-year after coronary artery bypass graft; SYNTAX = synergy between percutaneous coronary intervention with Taxus and cardiac surgery; T = ticagrelor.

a With nonpatency as outcome.

b With occlusion as outcome.

For comparison of occlusion outcome as sensitivity analysis, in the per-graft analysis, T+A also had lower odds of occlusion compared with aspirin alone in the HbA1c <6.5% subgroup (adjusted OR for occlusion: 0.40; 95% CI: 0.17-0.94) and in the ≥6.5% subgroup (adjusted OR for occlusion: 0.49; 95% CI: 0.18-1.31). There was no interaction effect between baseline HbA1c status and antiplatelet treatment (interaction *P* = 0.664). There were no significant differences in occlusion for ticagrelor alone vs aspirin alone in both subgroups (all *P* > 0.05) and no interaction effect (interaction *P* = 0.154). Similar results were observed in the per-patient analysis (Table 4).

### Cardiovascular events and bleeding outcomes in patients with HbA1c <6.5% vs ≥6.5%

Thirteen major adverse cardiovascular events (MACEs), defined as a composite of cardiovascular death, myocardial infarction, and stroke, were observed within 1 year after CABG, with 6 (2.6%) in the HbA1c <6.5% subgroup and 7 (4.1%) in HbA1c ≥6.5% subgroup, respectively. Overall, the incidence of MACE was relatively low for all randomized antiplatelet treatments in both subgroups. There were no significant differences between the baseline HbA1c <6.5% and ≥6.5% subgroups and between antiplatelet treatments (all *P* > 0.05) (Table 5).

**Table 5:** 1-Year Cardiovascular and Bleeding in Baseline HbA1c Subgroups

Events	HbA1c<6.5%					HbA1c≥6.5%					*P* Value
	T+A (n = 79)	T (n = 73)	A (n = 81)	Total (n = 233)	*P* Value	T+A (n = 59)	T (n = 60)	A (n = 53)	Total (n = 172)	*P* Value	HbA1c <6.5% vs ≥6.5%
MACE	0	2 (2.7)	4 (4.9)	6 (2.6)	0.124	2 (3.4)	1 (1.7)	4 (7.5)	7 (4.1)	0.260	0.409
Cardiovascular death	0	0	0	0		1 (1.7)	0	2 (3.8)	3 (1.7)		0.207
Myocardial infarction	0	1 (1.4)	2 (2.5)	3 (1.3)		1 (1.7)	0	0	1 (0.6)		
Stroke	0	1 (1.4)	2 (2.5)	3 (1.3)		0	1 (1.7)	2 (3.8)	3 (1.7)		
Bleeding	31 (39.2)	8 (11)	6 (7.4)	46 (19.8)	<0.001	15 (25.4)	10 (16.7)	5 (9.4)	30 (17.5)	0.082	0.632
Major	2 (2.5)	1 (1.4)	0	3 (1.3)	0.419	0	0	0	0	-	0.265
Minor	2 (2.5)	0	0	2 (0.9)	0.211	0	0	1 (1.9)	1 (0.6)	0.308	1.000
Minimum	28 (35.4)	7 (9.6)	6 (7.4)	41 (17.6)	<0.001	15 (25.4)	10 (16.7)	4 (7.5)	29 (16.9)	0.039	0.846

Values are n (%).

A = aspirin; MACE = major adverse cardiovascular events; T = ticagrelor.

The incidence of overall bleeding events was similar between the HbA1c <6.5% and ≥6.5% subgroups (19.8% vs 17.5%, *P* = 0.632). In both HbA1c subgroups, the incidence of bleeding, particularly minimal bleeding, was significantly different among the randomized antiplatelet treatments (T+A: 35.4% vs T: 9.6% vs A: 7.4% in the HbA1c <6.5% subgroup, *P* < 0.001; T+A: 25.4% vs T: 16.7% vs A: 7.5% in the HbA1c ≥6.5% subgroup; *P* = 0.039).

## Discussion

This post hoc study of the DACAB trial aimed to assess the influence of baseline HbA1c levels on early (1-year) vein graft patency after CABG and the effects of a more aggressive antiplatelet therapy (ticagrelor with or without aspirin vs aspirin alone) on 1-year vein graft patency in patients with HbA1c <6.5% vs ≥6.5%. In the per-graft analysis, compared with the HbA1c <6.5% subgroup, the HbA1c ≥6.5% subgroup showed worse patency (adjusted OR for nonpatency: 1.69; 95% CI: 1.08-2.64; *P* = 0.021) and nonocclusion (adjusted OR for occlusion: 1.70; 95% CI: 1.04-2.79; *P* = 0.034). The association was also observed when HbA1c was analyzed as a continuous variable. T+A showed a higher vein graft patency rate than A in both HbA1c <6.5% subgroup (adjusted OR for nonpatency: 0.34; 95% CI: 0.15-0.75) and ≥6.5% subgroup (adjusted OR for nonpatency: 0.45; 95% CI: 0.19-1.09), without an interaction effect (*P* for interaction = 0.035). While, T did not show improvement compared with A in both subgroups. The results indicated that higher baseline HbA1c was associated with lower vein graft patency at 1 year after CABG. T+A improved 1-year vein graft patency vs A in patients who underwent CABG, irrespective of baseline HbA1c.

Previous studies showed that DM was not associated with the 1-year vein graft patency after CABG, including the BARI (Bypass Angioplasty Revascularization Investigation)^19^ and PREVENT IV (Prevention of Autogenous Vein Graft Failure in Coronary Artery Bypass Procedures)^20^ trials. A meta-analysis by Antonopoulos et al.^8^ showed that DM was independently associated with early (1-year) vein graft occlusion, with an OR of 1.43 (95% CI: 1.25-1.65). Nevertheless, using DM alone can bias the results because patients with DM with well-controlled glycemia will display a better microvascular and macrovascular status than patients with DM with poorly controlled glycemia.^21^ In the same line of thought, patients without DM with poorly controlled glycemia but not overt DM might have poorer outcomes than those with well-controlled glycemia.^15^

Hence, using HbA1c, which represents the overall glycemic control over the past 3 months,^9^ might yield more precise results and allow for more personalized medicine through better risk assessment. Maintaining low HbA1c levels has been shown to decrease the incidence of diabetic microvascular and macrovascular complications^10^, ^11^, ^12^ and cardiovascular death in general.^14^ Of note, Abu Tailakh et al.^13^ showed that patients with DM with high baseline HbA1c (>7%) were at higher risk of long-term complications and mortality after CABG. In patients with type 1 DM, high baseline HbA1c was associated with higher risks of long-term MACEs and death.^22^ A meta-analysis showed that after coronary interventions, baseline HbA1c levels were associated with renal failure and myocardial infarction in patients with DM and with mortality and renal failure in those without DM.^15^ Nevertheless, this meta-analysis also highlighted the lack of high-quality evidence. Another meta-analysis showed that the baseline HbA1c status was associated with morbidity and mortality after CABG, irrespective of DM status.^23^ However, all these studies examined morbidity and mortality after CABG, but none specifically examined the patency of the grafts.

A previous small-scale study suggested no differences in patency between patients with HbA1c <6.5% and ≥6.5% (96.9% vs 99.2%, *P* = 0.037),^24^ but the number of events was probably too small for reliable analyses. In the present study, compared with the HbA1c <6.5% subgroup, the HbA1c ≥6.5% subgroup showed worse patency (adjusted OR for nonpatency: 1.69; 95% CI: 1.08-2.64) and nonocclusion (adjusted OR for occlusion: 1.70; 95% CI: 1.04-2.79). We hypothesized that different HbA1c thresholds may influence the results. Still, the present study showed similar results when using 6.5%, 7.0%, 7.5%, and 8.0% as the cutoff HbA1c level or when analyzing HbA1c as a continuous variable.

Currently, the internal mammary artery is the golden standard of CABG because of high long-term patency rates and improved survival compared with any other grafts.^25^ Besides, Radial Artery Database International Alliance study and RAPCO (Radial Artery Patency and Clinical Outcomes) trial showed that radial artery grafts have better long-term patency outcome than vein grafts, which was considered as the second-choice graft.^7^^,^^26^ Lorusso et al. demonstrated that arterial grafts in patients with DM post CABG appear to sustain their biological integrity despite of the poor glycemic statues, which may explain the long-term patency rate. However, the same effect was not observed in the vein grafts.^27^ In our study, the 1-year graft outcome was obviously better in artery grafts than in vein grafts, which was consistent with the previous findings. Besides, there was no difference in 1-year artery graft outcome between patients with HbA1c <6.5% and ≥6.5%. Thus, for patients with poor glycemic control, autologous arteries might be more appropriate as grafts for CABG, which would be a testable opinion in the future.

Our study found that, in both per-graft and per patient analysis, T+A significantly prevented vein graft failure (nonpatency or occlusion) at 1 year in the HbA1c <6.5% subgroup and in the ≥6.5% subgroups, and there was no interaction between the HbA1c control and antiplatelet therapy. It suggests that a more aggressive dual antiplatelet therapy is sufficient to counteract the potential negative effects of HbA1c on thrombosis-related vein graft failure. Recently, the POPular CABG (Effect of Ticagrelor On Saphenous Vein Graft Patency in Patients Undergoing Coronary Artery Bypass Grafting Surgery) trial did not observe a decreased vein graft occlusion rate with T+A vs aspirin alone at 1 year after CABG. The OR for occlusion was 0.9 (95% CI: 0.3-2.7) in DM and 1.5 (95% CI: 0.7-2.9) in non-DM, with an interaction *P* = 0.49. Several potential reasons might explain this inconsistency. First, 35.3% of the patients in the POPular CABG trial had permanently discontinued study medication during the 1-year follow-up, including 8.0% who received study medication for <60 days. In the DACAB trial, only 3.4% of the patients discontinued study medication ≥60 days. The compliance was different in the 2 trials. Second, 94.6% of the patients in the POPular CABG trial underwent on-pump CABG, whereas the proportion was only 24.2% in the DACAB trial. Off-pump CABG had a negative effect on vein graft patency.^28^ In a post hoc analysis of the DACAB trial, more benefits of DAPT on vein graft patency were observed in the off-pump subgroup than the on-pump subgroup.^29^ Of note, the 1-year vein graft occlusion rate in the off-pump subgroup of the POPular CABG trial was 28.9%, which was much higher than the rate (8.9%) in the on-pump subgroup. Third, in the primary ITT analysis of the DACAB trial, 6.6% of the patients (6.2% of vein grafts) without primary outcome assessment at 1 year after CABG were imputed as occlusion, although in the POPular CABG trial, 10.6% of the patients without primary outcome assessment at 1 year after CABG were excluded from the ITT analysis. This difference in dealing with the missing data led to a higher 1-year vein graft occlusion rate in the DACAB trial (14.5%) than in the POPular CABG trial (9.9%). If that 6.6% of patients without primary outcome assessment were also excluded from the ITT analysis, the 1-year vein graft occlusion rate would be 8.8% in the DACAB trial, which was similar to that in the POPular CABG trial. In the prematurely terminated TiCAB (Ticagrelor in Coronary Artery Bypass) trial, ticagrelor monotherapy showed no advantage in preventing MACE or major bleeding than aspirin,^30^ which was in line with our results.

This study has limitations. First, baseline HbA1c levels were missing for 95 of 500 patients. The exclusion of these patients may cause data deviation. Second, most patients underwent off-pump CABG, which represents the current clinical practice status in China. Therefore, the conclusions may not apply to patients undergoing on-pump CABG. Third, we did not collect the data of type 1 and type 2 DM in the DACAB trial. Thus, the graft outcomes of patients with type 1 or type 2 DM were not compared. Fourth, the long-term outcomes are not yet available. Of note, a 5-year follow-up extension study is currently ongoing (NCT03987373). Fifth, we only analyzed the baseline HbA1c levels. The fluctuations in HbA1c levels during the intervention may occur, which was not considered in this post hoc analysis. Finally, the present study was a post hoc analysis of a completed trial, and the study design did not allow any causality determination. Future studies might be longitudinal in design and allow determination of the causes of vein graft failure. In addition, the power analysis of the original trial did not take into account baseline HbA1c.

## Conclusions

In the DACAB trial, higher baseline HbA1c was associated with lower vein graft patency 1 year after CABG. T+A improved 1-year vein graft patency vs aspirin, irrespective of baseline HbA1c. Overall, multiple artery grafts might be a more appropriate choice for these patients with poor glycemic control.

Perspectives **COMPETENCY IN MEDICAL KNOWLEDGE:** The influence of baseline HbA1c levels and the interaction effect of antiplatelet therapy on vein graft outcomes post CABG remains unclear. Our subgroup analysis of the DACAB trial showed higher baseline HbA1c was associated with worse vein graft patency 1 year after CABG. Ticagrelor plus aspirin showed higher 1-year vein graft patency compared with aspirin alone, irrespective of baseline HbA1c.

**TRANSLATIONAL OUTLOOK:** For patients with poor baseline glycemic control, the vein graft failure was a matter of concern even in the early term. Compared with aspirin alone, ticagrelor plus aspirin-based dual antiplatelet therapy could improve the 1-year vein graft patency, irrespective of baseline HbA1c. Thus, artery grafts might be a more appropriate choice for these patients, which needs further validation.

## Funding Support and Author Disclosures

The DACAB was funded by AstraZeneca; this post hoc analysis was not. Dr Zhu has served as a speaker for AstraZeneca, Johnson & Johnson, Novartis, and Sanofi; and as an investigator on clinical trials sponsored by AstraZeneca, Bayer, Novartis, and Sanofi. Dr Liu has served as a speaker for Pfizer. Dr R. Wang has served as a speaker for AstraZeneca; and as an investigator on clinical trials sponsored by Bayer. Dr. X. Wang has served as a speaker for AstraZeneca and Johnson & Johnson. Dr Cheng has served as a speaker for Medtronic. Dr Zhao has served as a speaker for AstraZeneca, Johnson & Johnson, and Medtronic; and has been an investigator on clinical trials sponsored by AstraZeneca, Bayer, Novartis, and Sanofi. All other authors have reported that they have no relationships relevant to the contents of this paper to disclose.
