# Supplementary material for: Influence of Baseline HbA1c and Antiplatelet Therapy on 1-Year Vein Graft Outcome
Source: JACC Asia. 2022 Mar 15;2(2):197–206. doi: 10.1016/j.jacasi.2021.11.009 (PMC9627937; doi:10.1016/j.jacasi.2021.11.009)
Supplement: Supplemental Tables 1–5 [file mmc1.docx]

**Supplemental Table 1** Characteristics of the patients between baseline HbA1c ≥ 6.5% and <6.5% and antiplatelet treatment

| Characteristics | HbA1c<6.5% | | | HbA1c≥6.5% | | |
| --- | --- | --- | --- | --- | --- | --- |
|  | T+A  (n=79) | T alone  (n=73) | A alone  (n=81) | T+A  (n=59) | T alone  (n=60) | A alone  (n=53) |
| Age, mean ± SD, y | 63.8 ± 8.4 | 63.5 ± 8.3 | 64.0 ± 7.7 | 64.5 ± 7.5 | 63.6 ± 8.5 | 64.4 ± 8.2 |
| Male gender, n (%) | 65 (82.3) | 62 (84.9) | 71 (87.7) | 42 (71.2) | 48 (80.0) | 42 (79.3) |
| Clinical status, n (%) |  |  |  |  |  |  |
| Stable angina | 24 (30.4) | 27 (37.0) | 29 (35.8) | 26 (44.1) | 29 (48.3) | 16 (30.2) |
| Unstable angina | 52 (65.8) | 45 (61.6) | 49 (60.5) | 31 (52.5) | 28 (46.7) | 36 (67.9) |
| NSTEMI | 3 (3.8) | 1 (1.4) | 3 (3.7) | 2 (3.4) | 3 (5.0) | 1 (1.9) |
| NYHA classification, n (%) |  |  |  |  |  |  |
| III-IV | 30 (38.0) | 32 (43.8) | 27 (33.3) | 26 (44.1) | 17 (28.3) | 20 (37.7) |
| Medical history, n (%) |  |  |  |  |  |  |
| Myocardial infarction | 29 (36.7) | 29 (39.7) | 19 (23.5) | 17 (28.8) | 22 (36.7) | 15 (28.3) |
| Stroke | 13 (16.5) | 5 (6.9) | 14 (17.3) | 10 (17.0) | 4 (6.7) | 6 (11.3) |
| Hypertension | 56 (70.9) | 55 (75.3) | 62 (76.5) | 51 (86.4) | 47 (78.3) | 37 (69.8) |
| Hyperlipidemia ^a^ | 55 (69.6) | 52 (71.2) | 59 (72.8) | 39 (66.1) | 45 (75.0) | 39 (73.6) |
| Peripheral vascular disease | 14 (17.7) | 16 (21.9) | 13 (16.1) | 11 (18.6) | 10 (16.7) | 13 (24.5) |
| Smoking | 49 (62.0) | 35 (48.0) | 43 (53.1) | 24 (40.7) | 25 (41.7) | 28 (52.8) |
| COPD | 11 (13.9) | 5 (6.9) | 6 (7.4) | 7 (11.9) | 1 (1.7) | 4 (7.6) |
| LVEDD, median (Q1, Q3), mm | 49 (46, 54) | 50 (47, 54) | 49 (45, 52) | 49 (45, 52) | 47 (45, 51) | 50 (47, 54) |
| LVEF, median (Q1, Q3), % | 61 (57, 68) | 62 (58, 66) | 64 (59, 68) | 62 (57, 68) | 62 (58, 69) | 63 (54, 66) |
| SYNTAX score, n (%) |  |  |  |  |  |  |
| Low (0-22) | 11 (13.9) | 8 (11.0) | 14 (17.3) | 5 (8.5) | 5 (8.3) | 11 (20.8) |
| Intermediate (23-32) | 47 (59.5) | 40 (54.8) | 50 (61.7) | 34 (57.6) | 33 (55.0) | 33 (62.3) |
| High (≥33) | 21 (26.6) | 25 (34.3) | 17 (21.0) | 20 (33.9) | 22 (36.7) | 9 (17.0) |
| EuroSCORE, n (%) |  |  |  |  |  |  |
| Low (0-2) | 37 (46.8) | 27 (37.0) | 35 (43.2) | 23 (39.0) | 28 (46.7) | 23 (43.4) |
| Medium (3-5) | 26 (32.9) | 39 (53.4) | 39 (48.2) | 25 (42.4) | 23 (38.3) | 22 (41.5) |
| High (≥6) | 16 (20.3) | 7 (9.6) | 7 (8.6) | 11 (18.6) | 9 (15.0) | 8 (15.1) |
| Medication use, baseline, n (%) |  |  |  |  |  |  |
| Beta blocker | 72 (91.1) | 67 (91.8) | 74 (91.4) | 55 (93.2) | 56 (93.3) | 49 (92.5) |
| ACEI/ARB | 45 (57.0) | 45 (61.6) | 60 (74.1) | 34 (57.6) | 43 (71.7) | 33 (62.3) |
| Statins | 78 (98.7) | 70 (95.9) | 78 (96.3) | 54 (91.5) | 57 (95.0) | 52 (98.1) |
| Medication use, 1 year, n (%) |  |  |  |  |  |  |
| Beta blocker | 77 (97.5) | 70 (95.9) | 73 (90.1) | 56 (94.9) | 56 (93.3) | 50 (94.3) |
| ACEI/ARB | 36 (45.6) | 37 (50.7) | 48 (59.3) | 36 (61.0) | 36 (60.0) | 28 (52.8) |
| Statins | 76 (96.2) | 72 (98.6) | 81 (100.0) | 52 (88.1) | 58 (96.7) | 50 (94.3) |
| Surgical procedure |  |  |  |  |  |  |
| Pump use, n (%) | 15 (19.0) | 9 (12.3) | 11 (13.6) | 10 (17.0) | 9 (15.0) | 13 (24.5) |
| Total grafts, No. | 293 | 278 | 306 | 222 | 236 | 199 |
| Mean grafts/patient, No. | 3.7 | 3.8 | 3.8 | 3.8 | 3.9 | 3.8 |
| IMA use, n (%) | 64 (81.0) | 63 (86.3) | 65 (80.2) | 51 (86.4) | 51 (85.0) | 42 (79.2) |

T = ticagrelor; A = aspirin; SD = standard deviation; NSTEMI = non-ST elevation myocardial infarction; NYHA = New York Heart Association; LDL-C = low-density lipoprotein cholesterol; COPD = chronic obstructive pulmonary disease; LVEDD = left ventricular end-diastolic diameter; LVEF = left ventricular ejection fraction; SYNTAX = synergy between percutaneous coronary intervention with Taxus and cardiac surgery; EuroSCORE = logistic European System for Cardiac Operative Risk Evaluation; ACEI = angiotensin-converting enzyme inhibitor; ARB = angiotensin receptor blocker; IMA = Internal mammary artery.

^a^ defined as baseline LDL-C ≥1.8 mmol/L

**Supplemental Table 2** One-year vein graft outcome between baseline HbA1c ≥ 7% and <7%

| 1-year outcome | HbA1c <7% | HbA1c ≥7% | HbA1c ≥7% vs. <7%  Adjusted OR (95% CI) | *P* |
| --- | --- | --- | --- | --- |
| Per graft | N=802 | N=388 |  |  |
| Fitzgibbon grade A, n (%) | 683 (85.2) | 300 (77.3) |  |  |
| Fitzgibbon grade B, n (%) | 21 (2.6) | 17 (4.4) |  |  |
| Fitzgibbon grade O, n (%) | 98 (12.2) | 71 (18.3) |  |  |
| Patency (A), n (%) | 683 (85.2) | 300 (77.3) | 1.74 (1.11, 2.74) ^a^ | 0.017 |
| Non-occlusion (A+B) , n (%) | 704 (87.8) | 317 (81.7) | 1.61 (0.98, 2.66) ^b^ | 0.062 |
| Per patient | N=278 | N=127 |  |  |
| Fitzgibbon grade A, n (%) | 211 (75.9) | 79 (62.2) |  |  |
| Fitzgibbon grade B, n (%) | 12 (4.3) | 11 (8.7) |  |  |
| Fitzgibbon grade O, n (%) | 55 (19.8) | 37 (29.1) |  |  |
| Patency (A), n (%) | 211 (75.9) | 79 (62.2) | 1.99 (1.21, 3.28) ^a^ | 0.007 |
| Non-occlusion (A+B), n (%) | 223 (80.2) | 90 (70.9) | 1.67 (0.99, 2.81) ^b^ | 0.055 |

OR = odds ratio; CI = confidence interval. OR was adjusted for age, sex, medical history of hypertension and hyperlipidemia, SYNTAX score, target vessel distribution, antiplatelet therapy and statin use at 1-year after coronary artery bypass graft (CABG).

^a^ with non-patency (B+O) as outcome.

^b^ with occlusion (O) as outcome.

**Supplemental Table 3** One-year vein graft outcome between baseline HbA1c ≥ 7.5% and <7.5%

| 1-year outcome | HbA1c <7.5% | HbA1c ≥ 7.5% | HbA1c ≥7.5% vs. <7.5%  Adjusted OR (95% CI) | *P* |
| --- | --- | --- | --- | --- |
| Per graft | N=893 | N=297 |  |  |
| Fitzgibbon grade A, n (%) | 759 (85.0) | 224 (75.4) |  |  |
| Fitzgibbon grade B, n (%) | 25 (2.8) | 13 (4.4) |  |  |
| Fitzgibbon grade O, n (%) | 109 (12.2) | 60 (20.2) |  |  |
| Patency (A), n (%) | 759 (85.0) | 224 (75.4) | 1.85 (1.16, 2.95) ^a^ | 0.010 |
| Non-occlusion (A+B), n (%) | 784 (87.8) | 237 (79.8) | 1.79 (1.07, 2.98) ^b^ | 0.027 |
| Per patient | N=308 | N=97 |  |  |
| Fitzgibbon grade A, n (%) | 232 (75.3) | 58 (59.8) |  |  |
| Fitzgibbon grade B, n (%) | 15 (4.9) | 8 (8.3) |  |  |
| Fitzgibbon grade O, n (%) | 61 (19.8) | 31 (32.0) |  |  |
| Patency (A), n (%) | 232 (75.3) | 58 (59.8) | 2.32 (1.37, 3.93) ^a^ | 0.002 |
| Non-occlusion (A+B), n (%) | 247 (80.2) | 66 (68.1) | 2.02 (1.17, 3.50) ^b^ | 0.012 |

OR = odds ratio; CI = confidence interval. OR was adjusted for age, sex, medical history of hypertension and hyperlipidemia, SYNTAX score, target vessel distribution, antiplatelet therapy and statin use at 1-year after coronary artery bypass graft (CABG).

^a^ with non-patency (B+O) as outcome.

^b^ with occlusion (O) as outcome.

**Supplemental Table 4** One-year vein graft outcome between baseline HbA1c ≥8 % and <8%

| 1-year outcome | HbA1c <8% | HbA1c ≥ 8% | HbA1c ≥8% vs. <8%  Adjusted OR (95% CI) | P |
| --- | --- | --- | --- | --- |
| Per graft | N=962 | N=228 |  |  |
| Fitzgibbon grade A, n (%) | 813 (84.5) | 170 (74.6) |  |  |
| Fitzgibbon grade B, n (%) | 29 (3.0) | 9 (4.0) |  |  |
| Fitzgibbon grade O, n (%) | 120 (12.5) | 49 (21.5) |  |  |
| Patency (A) , n (%) | 813 (84.5) | 170 (74.6) | 1.87 (1.14, 3.06) ^a^ | 0.014 |
| Non-occlusion (A+B) , n (%) | 842 (87.5) | 179 (78.6) | 1.89 (1.11, 3.22) ^b^ | 0.019 |
| Per patient | N=330 | N=75 |  |  |
| Fitzgibbon grade A, n (%) | 246 (74.6) | 44 (58.7) |  |  |
| Fitzgibbon grade B, n (%) | 18 (5.5) | 5 (6.7) |  |  |
| Fitzgibbon grade O, n (%) | 66 (20.0) | 26 (34.7) |  |  |
| Patency (A), n (%) | 246 (74.6) | 44 (58.7) | 2.21 (1.25, 3.91) ^a^ | 0.007 |
| Non-occlusion (A+B), n (%) | 264 (80.1) | 49 (65.4) | 2.18 (1.21, 3.93) ^b^ | 0.009 |

OR = odds ratio; CI = confidence interval. OR was adjusted for age, sex, medical history of hypertension and hyperlipidemia, SYNTAX score, target vessel distribution, antiplatelet therapy and statin use at 1-year after coronary artery bypass graft (CABG).

^a^ with non-patency (B+O) as outcome.

^b^ with occlusion (O) as outcome.

**Supplemental Table 5** One-year artery graft outcome between baseline HbA1c ≥ 6.5% and <6.5%

| 1-year outcome | HbA1c <6.5% | HbA1c ≥ 6.5% | *P* |
| --- | --- | --- | --- |
| Per graft | N=191 | N=131 |  |
| Fitzgibbon grade A, n (%) | 185 (96.9) | 128 (97.7) |  |
| Fitzgibbon grade B, n (%) | 1 (0.5) | 1 (0.8) |  |
| Fitzgibbon grade O, n (%) | 5 (2.6) | 2 (1.5) |  |
| Patency (A), n (%) | 185 (96.9) | 128 (97.7) | 0.743 |
| Non-occlusion (A+B) , n (%) | 186 (97.4) | 129 (98.5) | 0.705 |
| Per patient | N=189 | N=131 |  |
| Fitzgibbon grade A, n (%) | 183 (96.8) | 128 (97.7) |  |
| Fitzgibbon grade B, n (%) | 1 (0.5) | 1 (0.8) |  |
| Fitzgibbon grade O, n (%) | 5 (2.7) | 2 (1.5) |  |
| Patency (A), n (%) | 183 (96.8) | 128 (97.7) | 0.742 |
| Non-occlusion (A+B), n (%) | 184 (97.3) | 129 (98.5) | 0.705 |
